# Immunohistochemical Detection, In Situ Distribution, and Comparison of Tuft Cells and Other Selected Components of Mucosal Immunity in Nasal Turbinates of Piglets Challenged with Rotavirus from Sows Fed Vitamin A-Deficient Diets with or Without Vitamin A Supplementation

**DOI:** 10.3390/vetsci13090987

**Published:** 2026-09-18

**Authors:** Kwonil Jung, Anastasia N. Vlasova, Linda J. Saif

**Affiliations:** 1 Center for Food Animal Health, Department of Animal Sciences, College of Food, Agricultural, and Environmental Sciences, The Ohio State University, Wooster, OH 44691, USA; vlasova.1@osu.edu; 2 Department of Veterinary Preventive Medicine, College of Veterinary Medicine, The Ohio State University, Columbus, OH 43210, USA

**Keywords:** pig, nasal cavity, tuft cell, immunohistochemistry, MAdCAM-1, CCL25, pIgR

## Abstract

Tuft cells are a rare type of epithelial cell that are essential to regulate innate and adaptive immune responses. Mucosal immunity is also composed primarily of IgA produced by B cells that is secreted onto mucosal surfaces as secretory IgA via the polymeric immunoglobulin receptor (pIgR). The homing of B cells in lymphoid organs is mediated by certain adhesion receptors, such as α4β7 and chemokine receptor 9 (CCR9), that bind to the specific vascular ligands, mucosal vascular addressin cell adhesion molecule 1 (MAdCAM-1) and chemokine ligand 25 (CCL25), respectively. It is unclear whether tuft cells, MAdCAM-1, CCL25, and pIgR are present or expressed in the nasal cavities of pigs. Our study aimed to develop immunohistochemistry (IHC) or immunofluorescence (IF) staining for the detection of tuft cells, MAdCAM-1, CCL25, pIgR, and IgA- or IgG-positive cells in porcine nasal turbinates. The tissues were acquired from the piglets of two different treatment groups in our previous studies that investigated the impact of oral vitamin A supplementation (VA) on the maternal immunity of vitamin A-deficient (VAD) sows and the passive protection of their piglets against rotavirus A (RVA): (1) VAD + VA + RVA and (2) VAD + RVA piglets. Our IHC staining revealed that low numbers of advillin-positive, bottle-shaped cells that morphologically resemble tuft cells were present in the mucosal epithelium and the mucous duct or glands. There were also moderate to large amounts of MAdCAM-1 and CCL25 in the endothelial cells lining the vascular structures present in the lamina propria of nasal turbinates and moderate to large amounts of pIgR in the turbinate mucosal epithelium. Moderate to high numbers of IgA- or IgG-positive cells were present in the lamina propria. There were no differences in IF- or IHC-positive scores or the positive cell numbers of all parameters tested between VAD + VA + RVA and VAD + RVA piglets, suggesting little or no effect of VA on the parameters tested. Our study demonstrated the use of IHC/IF staining methods for the detection of tuft cells, MAdCAM-1, CCL25, pIgR, and IgA- or IgG-positive cells in porcine nasal turbinates, expanding our understanding of the in situ distribution and comparison in the nasal cavity of pigs born to the sows fed vitamin A-deficient diets with or without vitamin A supplementation.

## Simple Summary

We developed immunohistochemistry (IHC) or immunofluorescence (IF) staining for the detection of tuft cells and other selected components of mucosal immunity—MAdCAM-1, CCL25, pIgR, and IgA or IgG antibody-positive cells—in porcine nasal turbinates. The archival tissues were acquired from piglets of two different treatment groups in our previous studies that investigated the impact of oral vitamin A supplementation (VA) on the maternal immunity of vitamin A-deficient (VAD) sows and the passive protection of their piglets against rotavirus A (RVA): (1) VAD + VA + RVA and (2) VAD + RVA piglets. The IHC/IF staining methods we developed successfully detected and revealed the presence of tuft cells, MAdCAM-1, CCL25, pIgR, and IgA- or IgG-positive cells in porcine nasal turbinates with high similarity to the in situ distribution and other common features in other mucosal sites, such as the intestine. We also found no differences in IF- or IHC-positive scores or positive cell numbers of all parameters tested between VAD + VA + RVA and VAD + RVA piglets, suggesting little or no effect of VA on the parameters tested. The IHC/IF staining methods and antibodies are useful for the detection of tuft cells and other selected mucosal immune components in porcine turbinates and possibly other organs.

## 1. Introduction

Tuft cells are a rare type of epithelial cell in the intestinal and respiratory tracts of humans and mice [1]. The cell morphology is distinctive because of their microvilli that form a tuft-shaped, cytoplasmic extension. Despite their rarity, tuft cells are essential to regulate innate and adaptive immune responses by producing interleukin-25 and promoting type 2 immune responses in viral and parasitic infections [1]. In the nasal cavity of humans and mice, tuft cells are also known to produce acetylcholine to maintain epithelial homeostasis and mucociliary clearance [1]. Mucosal humoral immunity is largely composed of IgA and fewer IgG antibodies produced by mucosal B cells. The homing of intestinal or mucosal T or B cells to secondary lymphoid organs and their trafficking in gut-associated lymphoid tissues (GALTs) is mediated by different chemokines or cell adhesion receptors expressed on the cellular surface and the specific ligands present within distinct vessels or high endothelial venules. Those include mucosal vascular addressin cell adhesion molecule 1 (MAdCAM-1) and chemokine ligand 25 (CCL25) [2,3], which interact with cell adhesion receptors α4β7 and chemokine receptor 9 (CCR9), respectively, expressed on the surface of mucosal T or B cells [2,3,4]. Previous studies in mice, humans, and pigs indicate that MAdCAM-1 and CCL25 are mostly confined to the small intestine [2,5,6]. Our previous study in pigs also identified the presence of MAdCAM-1 and CCL25 in the vascular structures of the mammary gland [7]. The lamina propria of the intestine contains polymeric immunoglobulins (pIgs) produced by mucosal B cells, such as dimeric IgA or pentameric IgM. The pIgs are present at the basement membrane of enterocytes and then transported across the epithelium via the polymeric immunoglobulin receptor (pIgR) expressed on the apical and/or basolateral surface of enterocytes [8,9]. Previous studies reported the presence of pIgR in the crypts of the small intestine and the acini of the mammary gland of pigs [7,9]. It is unclear whether or to what extent tuft cells, MAdCAM-1, CCL25, and pIgR are present or expressed in the nasal cavities of pigs.

Mucosal innate or humoral immunity in the nasal cavity is essential for the neutralization of upper respiratory tract pathogens such as seasonal influenza A, the interference of pathogen–cell attachment, and the clearance of the infection [10]. The tuft cells, MAdCAM-1, CCL25, pIgR, and IgA- or IgG-positive cells are essential components of the mucosal immunity in the nasal cavity. The protocols and reagents for the in situ detection of these mucosal immune components in the nasal cavities of pigs have not previously been described or developed. Our study aimed to develop immunohistochemistry (IHC) or immunofluorescence (IF) staining for the detection of tuft cells and other selected mucosal immune components, MAdCAM-1, CCL25, pIgR, and IgA- or IgG-positive cells in the nasal turbinates of pigs using archival formalin-fixed, paraffin-embedded tissues acquired from our previous studies [11]. In our prior studies, we investigated the impact of oral vitamin A supplementation (VA) on the maternal immunity of vitamin A-deficient (VAD) sows and passive protection of their piglets against rotavirus A (RVA). We used the two different VA treatment groups: (1) VAD + VA + RVA and (2) VAD + RVA piglets born to the sows fed VAD diet ± oral VA. In addition to the identification, characterization, and distribution of IF- or IHC-positive cells, we also investigated possible differences in IF- or IHC-positive scores or positive cell numbers between the two different treatment groups of piglets because the dam of VAD + VA + RVA piglets previously showed enhanced maternal adaptive immunity as a possible impact of oral VA. For example, VAD decreased memory B cell frequencies while VA supplementation increased RVA-specific IgA/IgG antibody-secreting cell numbers in the blood, milk, and tissues of RVA-inoculated VAD sows, suggesting a role of VA in B cell immunity and trafficking to tissues [11].

## 2. Materials and Methods

### 2.1. Archival Nasal Turbinate Tissues

The nasal turbinate tissue samples tested in our study were archival formalin-fixed, paraffin-embedded tissues acquired from the piglets in our previous studies [11]. Briefly, four rotavirus seropositive sows were fed VAD diets starting at approximately gestation day (GD) 30. Two of four VAD sows (VAD + VA) were given daily oral retinyl palmitate (30,000 IU) starting at approximately GD 76 throughout the rest of gestation and lactation. All four sows were inoculated orally with 1 × 10^9^ FFU of RVA strain OSU G5[P7] at GD 84–93 [VAD + VA + RVA sows (*n* = 2); VAD + RVA sows (*n* = 2)]. Their 3–5-day-old piglets were also inoculated orally with 1 × 10^8^ FFU of the same rotavirus. The piglets were euthanized at approximately post-inoculation day (PID) 14. The intestinal tissues, liver, and blood were previously collected to investigate the impact of oral VA in the maternal immunity of VAD sows and the passive protection of their piglets against RVA [11]. In addition, the nasal turbinate (or anatomically, nasoturbinate [12]) tissues were also collected from three pigs per sow [VAD + VA + RVA piglets (*n* = 6); VAD + RVA piglets (*n* = 6)] at PID 14. All tissues were fixed in 10% neutral buffered formalin for 2 days prior to decalcification. The Institutional Animal Care and Use Committee (IACUC) of The Ohio State University approved all protocols related to the animal experiments in this study (protocol # 2015A00000071).

### 2.2. Histological Evaluation and RNAscope In Situ Hybridization for the Detection of Rotavirus A Virus RNA in Porcine Nasal Turbinate Tissues

After decalcification in 10%-buffered EDTA (pH 7.4) in phosphate-buffered saline (PBS), the formalin-fixed, nasal turbinate tissues were embedded in paraffin, sectioned (3.5 μm), and evaluated by hematoxylin and eosin staining and tested by RNAscope in situ hybrization (ISH) using the RNAscope 2.5 HD detection kit (Advanced Cell Diagnostics, Newark, CA, USA; cat. no. 322300) and probe (Advanced Cell Diagnostics; cat. no. 447231) targeting the viral protein 6 gene for the detection of RVA RNA as described previously [13]. The archival formalin-fixed, paraffin-embedded intestinal tissue of a gnotobiotic pig infected previously with RVA was used as a positive control.

### 2.3. IHC Staining for the Detection of Tuft Cells in Porcine Nasal Turbinate Tissues

The tuft cell marker advillin, identified previously in the intestinal tuft cells of mice [14], was tested to detect tuft cells in porcine nasal turbinate tissues. For the IHC of advillin-positive tuft cells, a proteolytic-induced antigen retrieval using proteinase K (Invitrogen; 20 mg/ml; cat. no. 25530049) was used. The tissues were incubated in a polyclonal rabbit antibody against human advillin (Proteintech, Rosemont, IL, USA; cat. no. 20956-1-AP) diluted (1:100) in PBS at 4 °C overnight. Goat anti-rabbit antibody (diluted 1:50 in PBS) conjugated with horseradish peroxidase (Invitrogen, Carlsbad, CA, USA; cat. no. 65-6120) was used as the secondary antibody. The secondary antibody alone-incubated tissues were used as a control of IHC staining. The mean numbers of advillin-positive tuft cells were estimated by directly counting IHC-positive cells in four different microscopic areas (×200) of a nasal turbinate section of each pig, and the mean (±standard deviation) of all group pigs’ values was calculated. Advillin antigens were visualized as brown staining using a DAB substrate kit (Vector Laboratories, Newark, CA, USA; cat. no. SK-4100).

### 2.4. IF Staining for the Detection of MAdCAM-1, CCL25, and pIgR in Porcine Nasal Turbinate Tissues

For the IF staining of MAdCAM-1, CCL25, or pIgR antigen, a heat-induced antigen retrieval using a microwave and 0.01 M citric acid (pH 6.0) was used as described previously [7]. Two monoclonal antibodies against human MAdCAM-1 (Novus biologicals, Centennial, CO, USA; cat. no. NBP2-45706) or CCL25 (Novus biologicals; cat. no. MAB3341) and a polyclonal rabbit antibody against human pIgR (Proteintech; cat. no. 22024-1-AP) diluted 1:100 in PBS were incubated on the tissues at 4 °C overnight. Goat anti-mouse (Invitrogen; cat. no. A11017) or anti-rabbit antibody (Invitrogen; cat. no. A11034) (diluted 1:200 in PBS) conjugated with Alex Fluor^®^488 was used as the secondary antibody. The secondary antibody alone-incubated tissues were used as a control of IF staining. The MAdCAM-1 or CCL25 antigen-positive score per group was evaluated based on the mean number and intensity of IF-positive cells in three different microscopic areas (×200) of a nasal turbinate section of each pig, as follows: 0, no positive vessels showed staining; 1, low numbers of positive vessels showed mild staining; 2, moderate numbers of positive vessels showed moderate staining; and 3, high numbers of positive vessels showed strong staining. The pIgR antigen-positive score per group was evaluated based on the mean number and intensity of IF-positive cells in three different microscopic areas (×200) of a nasal turbinate section of each pig, as follows: 0, no positive cells showed staining; 1, low numbers of positive cells showed mild staining; 2, moderate numbers of positive cells showed moderate staining; and 3, high numbers of positive cells showed strong staining.

### 2.5. IHC/IF Staining for the Detection of IgA- or IgG-Positive Cells in Porcine Nasal Turbinate Tissues

For IHC or IF of IgA- or IgG-positive cells, a proteolytic-induced antigen retrieval using proteinase K (Invitrogen; 20 mg/ml) was used as described previously [7]. A polyclonal goat anti-pig IgA antibody conjugated with horseradish peroxidase (Bio-RAD, Hercules, CA, USA; cat. no. AAI40P) and a polyclonal goat anti-pig IgG (H + L) antibody conjugated with fluorescein (Seracare, Milford, MA, USA; cat. no. 5230-0297) diluted 1:100 in PBS were incubated onto the tissues at 4 °C overnight. The archival formalin-fixed, paraffin-embedded splenic tissues of two Severe Combined Immunodeficiency (SCID) gnotobiotic pigs, deficient in B and T cells, were used as a negative control. The IgA- or IgG-positive score per group was evaluated based on the mean number of IHC- or IF-positive cells in three different microscopic areas (×200) of a nasal turbinate section of each pig, as follows: 0 (negative), no positive cells; 1 (low), less than 10 IHC- or IF-positive cells; 2 (moderate), 10 to 20 IHC- or IF-positive cells; and 3 (high), more than 20 IHC- or IF-positive cells. All IF stained slides were evaluated under a fluorescence microscopy (Olympus, Tokyo, Japan), whereas IgA antigens were visualized as brown staining using a DAB substrate kit (Vector Laboratories).

### 2.6. Data Analysis

All values are expressed as the means ± standard deviation of the means (SDM). The IHC- or IF-positive scores or positive cell numbers from all pigs were analyzed and compared by Student’s *t*-test using GraphPad Prism 8 (GraphPad Prism Inc., San Diego, CA, USA). A value of *p* < 0.05 was considered statistically significant.

## 3. Results

### 3.1. Clinical Observations, Hepatic Vitamin A Level, RVA RNA Detection in the Pigs Used, and Histology and ISH Results of the Archival Nasal Turbinate Tissues

After RVA inoculation, the clinical disease was limited to the gastrointestinal tract and mainly consisted of loose stools to watery diarrhea. The detailed clinical disease, fecal virus shedding, and hepatic vitamin A level in RVA-inoculated, VAD + VA + RVA- or VAD + RVA-group pigs, which were born to sows fed vitamin A-deficient diet ± vitamin A supplementation, were reported in our previous paper [11]. Briefly, the prior studies found that the sows fed VA-deficient diets (VAD) had decreased hepatic VA levels compared with VAD + VA sows (supplemented VA start at approximately GD 76) at approximately day post-partum 21 (around PID 14). In their offspring, concomitantly, there were significantly lower hepatic vitamin A levels in VAD + RVA-group pigs compared with VAD + VA + RVA-group pigs. The prior studies also found low and compatible RVA RNA shedding titers in rectal swabs of the VAD + VA + RVA- and VAD + RVA-group pigs at PID 1 to 12, with no statistical differences in the mean cumulative fecal consistency scores or fecal RVA RNA titers between the two groups. At euthanasia (PID 14), little or no viral RNA was found in the feces of the pigs regardless of the treatment group [11]. None of twelve VAD + VA + RVA- or VAD + RVA-group pigs tested had histological abnormalities in the nasal turbinate tissues (Figure S1A,B in the Supplementary Materials). None of twelve VAD + VA + RVA- or VAD + RVA-group pigs tested had RVA RNA-positive cells in the nasal turbinate tissues by ISH (Figure S2A,C in the Supplementary Materials).

### 3.2. Detection of Advillin-Positive Tuft Cells by IHC Staining in Porcine Nasal Turbinate Tissues

**Figure 1:**
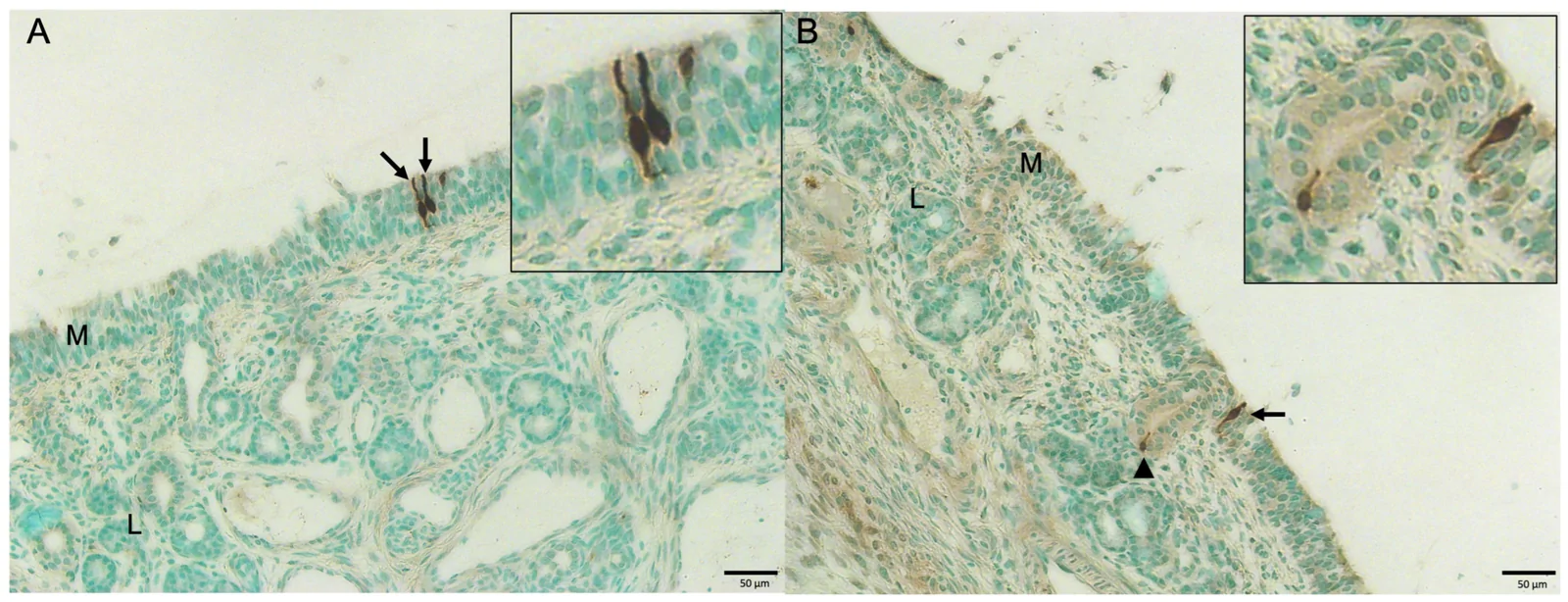
Detection of advillin-positive tuft cells by immunohistochemistry (IHC) in porcine nasal turbinate tissues. (**A**) IHC-stained turbinate of a pig from VAD + VA + RVA group at post-inoculation day (PID) 14, showing a low number of advillin-positive tuft cells (dark brown color; arrows) in the turbinate epithelium. Inset—Note two bottle-shaped tuft cells with discrete apical and/or basal cytoplasmic extensions. (**B**) IHC-stained turbinate of a pig from VAD + RVA group at PID 14, showing a low number of advillin-positive tuft cells (brown color) in the turbinate epithelium (arrow) or mucous duct (arrowhead). Inset—Note bottle-shaped tuft cells with discrete apical and/or basal cytoplasmic extensions in the turbinate epithelium or mucous duct. Scale bars = 50 µm. DAB substrate and methyl green counterstaining. M, mucosal epithelium; L, lamina propria.

By IHC staining, DAB substrate-visualized, advillin-positive tuft cells were dark brown and morphologically bottle-shaped with discrete apical and/or basal cytoplasmic extensions or occasionally round-shaped (Figure 1A,B). Both the nucleus and cytoplasm of cells were dark brown-colored. Under our IHC staining conditions, the turbinates of VAD + VA + RVA- or VAD + RVA-group pigs (15–17-day-old) at PID 14 contained low numbers of advillin-positive tuft cells in the mucosal epithelium (Figure 1A,B and Table 1). In the lamina propria, occasionally, a few cells lining the mucous duct or glands were also positive for advillin (Figure 1B and Figure S3 in the Supplementary Materials). Mean numbers (±SDM) of advillin-positive tuft cells in the turbinate mucosal epithelium of VAD + VA + RVA- and VAD +RVA-group pigs at PID 14 were 4.0 (±2.6) and 2.8 (±2.8), respectively (Table 1). Mean numbers (±SDM) of advillin-positive tuft cells in the turbinate lamina propria (glandular epithelium) of VAD + VA + RVA- and VAD + RVA-group pigs at PID 14 were 0.4 (±1.1) and 0.3 (±0.8), respectively. By Student’s *t*-test, the mean numbers of advillin-positive tuft cells in the turbinate mucosal epithelium or lamina propria (glandular epithelium) of VAD + VA + RVA- and VAD + RVA-group pigs at PID 14 did not differ significantly.

### 3.3. Detection of MAdCAM-1 and CCL25 by IF Staining in Porcine Nasal Turbinate Tissues

**Figure 2:**
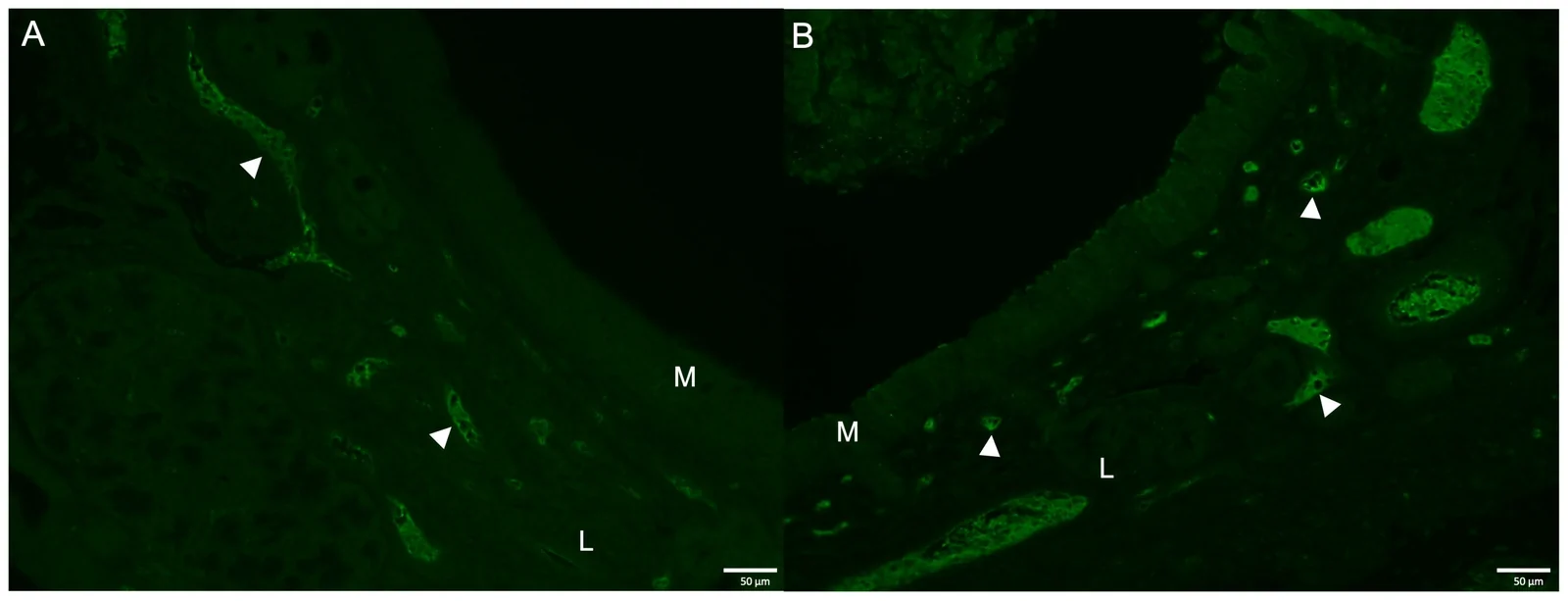
Detection of MAdCAM-1 (**A**) and CCL25 (**B**) by immunofluorescence (IF) staining in porcine nasal turbinate tissues. (**A**) IF-stained turbinate of a pig from VAD + VA + RVA group at post-inoculation day (PID) 14 showing a moderate to large amount of MAdCAM-1 (green color) in the endothelial cells lining the vascular structures (arrowheads) present in the lamina propria. (**B**) IF-stained turbinate of a pig from VAD + VA + RVA group at PID 14 showing a large amount of CCL25 (green color) in the endothelial cells lining the vascular structures (arrowheads) present in the lamina propria. Scale bars = 50 µm. M, mucosal epithelium; L, lamina propria.

Under our IF staining conditions, the turbinates of VAD + VA + RVA- or VAD + RVA-group pigs (15–17 days old) at PID 14 contained moderate to large amounts of MAdCAM-1 or CCL25 in the endothelial cells lining the vascular structures present in the lamina propria (Figure 2A,B, and Table 1). No MAdCAM-1- or CCL25-positive cells were detected in the mucosal epithelium of the nasal turbinates (Table 1). Mean (±SDM) MAdCAM-1-positive scores in the turbinates of VAD + VA + RVA- and VAD + RVA-group pigs at PID 14 were 2.4 (±0.4) and 2.3 (±0.8), respectively. Mean (±SDM) CCL25-positive scores in the turbinate endothelium of VAD + VA + RVA- and VAD + RVA-group pigs at PID 14 were 2.6 (±0.4) and 2.4 (±0.5), respectively. By Student’s *t*-test, MAdCAM-1- or CCL25-positive scores in the turbinates of VAD + VA + RVA- and VAD + RVA-group pigs at PID 14 did not differ significantly.

### 3.4. Detection of pIgR by IF Staining in Porcine Nasal Turbinate Tissues

**Figure 3:**
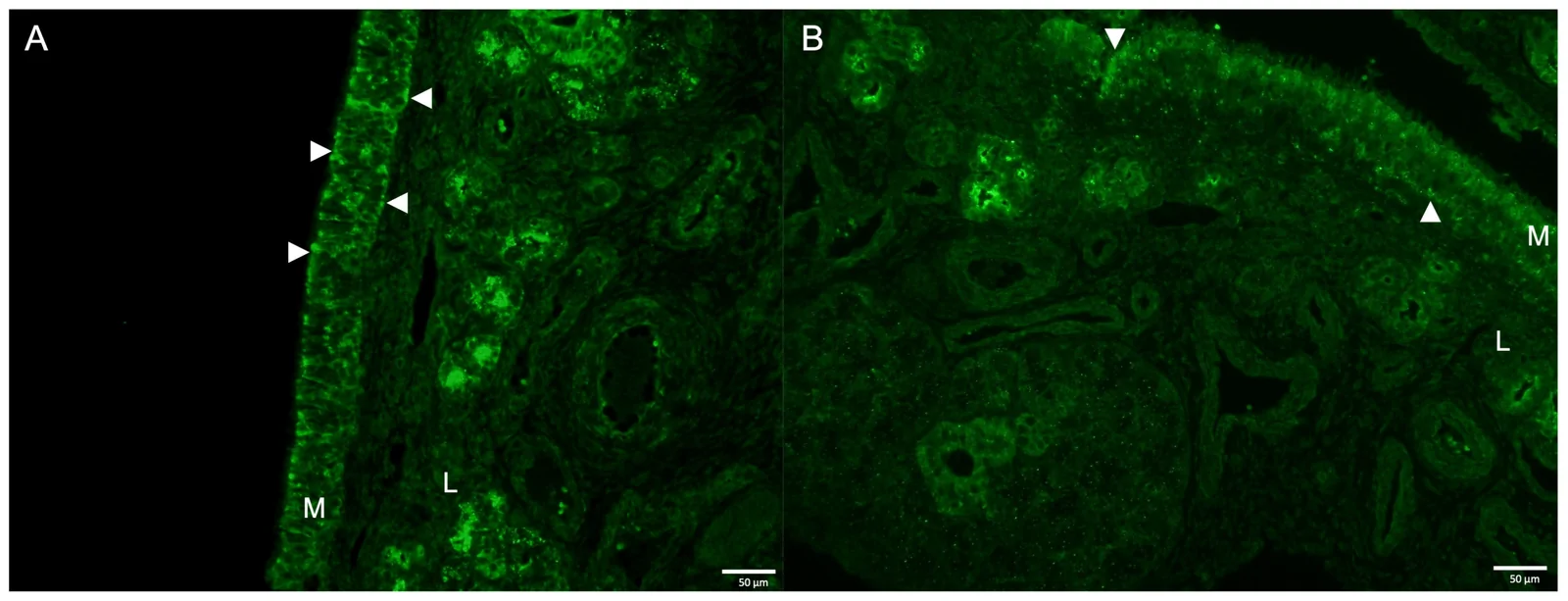
Detection of pIgR by immunofluorescence (IF) staining in porcine nasal turbinate tissues. IF-stained turbinates of a pig from VAD + RVA group (**A**) and a pig from VAD + VA + RVA group (**B**) at post-inoculation day 14 showing moderate to large amounts of pIgR (green color) in the epithelial cells lining the turbinate mucosal epithelium or forming the mucous glands. Notice that pIgR is detected on the apical and/or basal surface (arrowheads) of the epithelial cells lining the turbinate mucosal epithelium. Scale bars = 50 µm. M, mucosal epithelium; L, lamina propria.

Under our IF staining conditions, the turbinates of VAD + VA + RVA- or VAD + RVA-group pigs (15–17 days old) at PID 14 contained moderate to large amounts of pIgR in the epithelial cells lining the turbinate mucosal epithelium or forming the mucous glands (Figure 3A,B, and Table 1). Notably, pIgR was detected on the apical and/or basal surface of the epithelial cells lining the turbinate mucosal epithelium (Figure 3A,B). Mean (±SDM) pIgR-positive scores in the turbinate mucosal epithelium of VAD + VA + RVA- and VAD +RVA-group pigs at PID 14 were 2.1 (±0.3) and 2.1 (±0.5), respectively (Table 1). Mean (±SDM) pIgR-positive scores in the lamina propria (glandular epithelium) of VAD + VA + RVA- and VAD + RVA-group pigs at PID 14 were 2.3 (±0.4) and 2.2 (±0.5), respectively. By Student’s *t*-test, pIgR-positive scores in the turbinate mucosal epithelium or lamina propria (glandular epithelium) of VAD + VA + RVA- and VAD + RVA-group pigs at PID 14 did not differ significantly.

### 3.5. Detection of IgA-Positive Cells by IHC Staining in Porcine Nasal Turbinate Tissues

**Figure 4:**
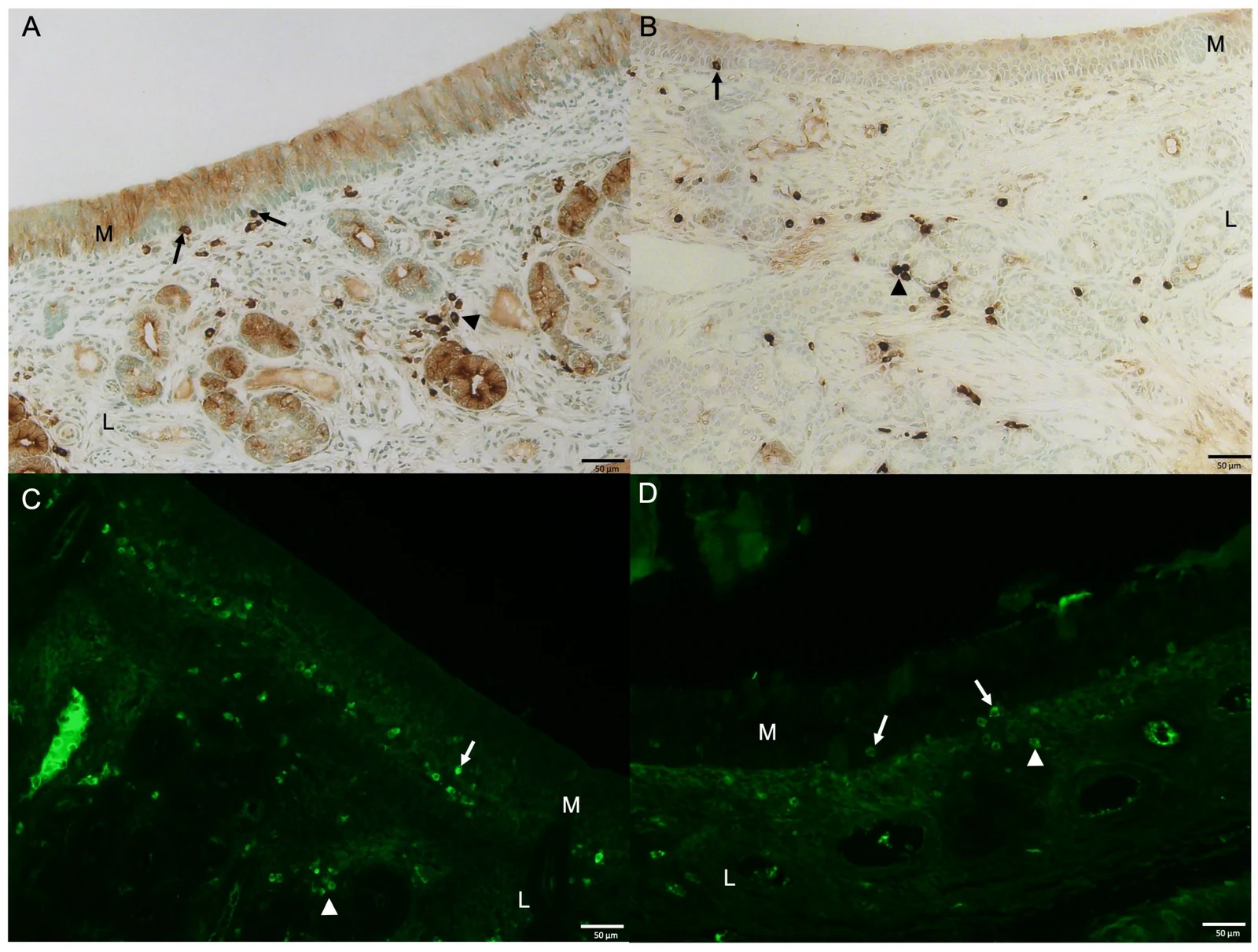
Detection of IgA- (**A**,**B**) or IgG-positive cells (**C**,**D**) by immunohistochemistry (IHC) or immunofluorescence (IF) staining in porcine nasal turbinate tissues. IHC-stained turbinate of a pig from VAD + VA + RVA group (**A**) and a pig from VAD + RVA group (**B**) at post-inoculation day (PID) 14, showing moderate to high numbers of IgA-positive cells (brown color) in the lamina propria, especially, the peri-glandular regions (arrowhead). Notice a few IgA-positive cells in the basal layer of the turbinate epithelium (arrows). IF-stained turbinate of a pig from VAD + VA + RVA group (**C**) and a pig from VAD + RVA group (**D**) at PID 14, showing moderate to high numbers of IgG-positive cells (green) in the lamina propria (arrowhead). Notice a few IgG-positive cells in the basal layer of the turbinate epithelium (arrows). Scale bars = 50 µm. (**A**,**B**), DAB substrate and methyl green counterstaining. M, mucosal epithelium; L, lamina propria.

By our IHC staining, DAB substrate-visualized IgA-positive cells were dark brown and morphologically round, oval to occasionally elongate. Under our IHC staining conditions, the turbinates of VAD + VA + RVA- or VAD + RVA-group pigs (15–17 days old) at PID 14 contained moderate to high numbers of IgA-positive cells in the lamina propria, especially the peri-glandular regions (Figure 4A,B, and Table 1), whereas no IgA-positive cells were detected in the spleens of two SCID gnotobiotic pigs co-tested as a negative control (Figure S4A in the Supplementary Materials). A few IgA-positive cells were also detected in the basal layer of the turbinate epithelium (Figure 4A,B). Mean (±SDM) IgA-positive scores in the turbinate mucosal epithelium of VAD + VA + RVA- and VAD + RVA-group pigs at PID 14 were 0.1 (±0.3) and 0.2 (±0.4), respectively (Table 1). Mean (±SDM) IgA-positive scores in the turbinate lamina propria of VAD + VA + RVA- and VAD + RVA-group pigs at PID 14 were 2.6 (±0.6) and 2.5 (±0.5), respectively (Table 1). By Student’s *t*-test, IgA-positive scores in the turbinate mucosal epithelium or lamina propria of VAD + VA + RVA- and VAD + RVA-group pigs at PID 14 did not differ significantly.

### 3.6. Detection of IgG-Positive Cells by IF Staining in Porcine Nasal Turbinate Tissues

By IF staining, IgG-positive cells were green and morphologically round, oval to occasionally elongate. Under our IF staining conditions, the turbinates of VAD + VA + RVAs or VAD + RVA-group pigs (15–17 days old) at PID 14 contained moderate to high numbers of IgG-positive cells in the lamina propria (Figure 4C,D, and Table 1), whereas no IgG-positive cells were detected in the spleens of two SCID gnotobiotic pigs co-tested as a negative control (Figure S4B in the Supplementary Materials). A few IgG-positive cells were also detected in the basal layer of the turbinate epithelium (Figure 4C,D). Mean (±SDM) IgG-positive scores in the turbinate mucosal epithelium of VAD + VA + RVA- and VAD + RVA-group pigs at PID 14 were 0.3 (±0.3) and 0.2 (±0.3), respectively (Table 1). Mean (±SDM) IgG-positive scores in the turbinate lamina propria of VAD + VA + RVA- and VAD + RVA-group pigs at PID 14 were 2.6 (±0.5) and 2.5 (±0.6), respectively (Table 1). By Student’s *t*-test, IgG-positive scores in the turbinate mucosal epithelium or lamina propria of VAD + VA + RVA- and VAD + RVA-group pigs at PID 14 did not differ significantly.

## 4. Discussion

Our study demonstrates that the monoclonal or polyclonal antibodies tested are useful for the detection of MAdCAM-1, CCL25, pIgR, IgA- or IgG-secreting cells and particularly for advillin-positive tuft cells in porcine turbinates and possibly other organs. Indeed, most of the antibodies were also useful to detect MAdCAM-1, CCL25, pIgR, and IgA- or IgG-secreting cells in porcine mammary glands [7]. Only brief materials and methods tested for the mammary gland were described previously [7]. In the current study, we attempted to further test or detail various combinations of proteolytic- vs. heat-induced antigen retrieval methods along with different primary (e.g., monoclonal vs. polyclonal antibodies for pIgR) or secondary (enzyme- vs. fluorescence-conjugated) antibodies of various concentrations. The optimal staining conditions of MAdCAM-1, CCL25, pIgR, IgA- or IgG-positive cells, or advillin-positive tuft cells in formalin-fixed, paraffin-embedded nasal turbinates of pigs are fully described in Section 2. The staining methods and antibodies can be used to investigate whether or to what extent the selected components of mucosal immunity—particularly, the chemoattaractant CCL25 for monocytes and macrophages—are involved in the pathogenesis of respiratory viruses causing interstitial pneumonia, such as porcine reproductive and respiratory virus, in the upper and lower respiratory tract of pigs, as well as to what extent they correlate with mucosal B cell responses after nasal vaccination against respiratory viruses. However, our study has several limitations. Our study did not include a mock control group in comparison with the VAD + VA + RVA- and VAD + RVA-group pigs born to the sows fed VAD diet ± oral VA and subsequently exposed to RVA. For the study, there was a limited availability of mock sows fed a normal diet and offspring unexposed to RVA. The selected immune components tested also represent only a limited part of mucosal immunity. The number of animals per group (*n* = 6) may be too small to detect subtle differences between the two treatment groups.

In the nasal cavitites of humans or mice, tuft cells are largely known to: (1) stimulate neighboring ciliated epithelial cells to secrete antimicrobial peptides, such as beta defensins 1 and 2; (2) produce acetylcholine to maintain epithelial homeostasis and mucociliary clearance; and (3) regulate innate and adaptive immune responses by producing interleukin-25 and promoting type 2 immune responses in viral and parasitic infections [1]. Although a low number of tuft cells were identified in the intestinal and respiratory tracts of humans or mice [1,14,15], it is unclear whether they are also present in the nasal turbinates of pigs. Our study clearly demonstrated the presence of low numbers of advillin-positive tuft cells in the mucosal or glandular epithelium of porcine nasal turbinate. Under our IHC staining conditions, the majority of advillin-positive tuft cells in the mucosal epithelium were morphologically bottle-shaped and had discrete apical and/or basal cytoplasmic extensions, corresponding to the cell morphology of tuft cells identified in the intestinal or respiratory tracts of humans or mice [1,14,15]. A few IHC-positive cells were also found in the bronchial or small intestinal epithelium of these piglets. Similar to intestinal tuft cells in mice, porcine turbinate tuft cells also appeared to express advillin protein, which might be useful as the cell marker of porcine tuft cells [14]. In our study, mean numbers of advillin-positive tuft cells in the nasal turbinate tissues of VAD + VA + RVA- and VAD + RVA-group pigs did not differ significantly (Table 1), suggesting few or no effects of VA on the number of tuft cells in porcine nasal turbinates. Under the same IHC staining condition for advillin-positive porcine turbinate tuft cells, it is noteworthy that there were no human advillin-positive cells in the bovine intestinal (from a germfree calf) or chicken intestinal (from 3-week-old conventional chickens) tissues tested along with the porcine nasal turbinates, indicating little or no possible cross-reaction of the primary antibody with bovine or avian tissues.

Previous studies in mice, humans, and pigs indicated that MAdCAM-1 and CCL25, essential for effectively recruiting α4β7- and/or CCR9-positive lymphocytes (predominantly, T cells and fewer B cells), are mostly confined to the small intestine [2,3,5,9]. In addition to the intestine, our study revealed that moderate to large amounts of MAdCAM-1 and CCL25 are also present in the nasal turbinates of pigs. Both MAdCAM-1 and CCL25 were similarly expressed in the endothelial cells lining the vascular structures present in the lamina propria. In addition to the presence of pIgR in the small intestine of pigs [9], our study found moderate to large amounts of pIgR in the epithelial cells lining the turbinate mucosal epithelium or the glandular epithelium. Similar to the anatomic location of mononuclear cells such as lymphocytes in the intestinal lamina propria of pigs, IgA- or IgG-positive cells were also predominantly present in the lamina propria of nasal turbinates, occasionally alongside a few intraepithelial IgA- or IgG-positive cells (Figure 3A,B).

The homing of intestinal IgA-secreting B cells to secondary lymphoid organs and their trafficking in GALTs are mediated by the cell adhesion receptors expressed on the cellular surface, such as α4β7 and CCR9 that interact and bind to the specific ligands MAdCAM-1 and CCL25, respectively [2]. The number of IgA-secreting B cells may positively correlate with the amount and density of these endothelial surface molecules in the intestine and other mucosal tissues, such as the nasal turbinates. The vitamin A (retinol) metabolite retinoic acid plays a fundamental role in the gut-specific homing of lymphocytes, such as T and B cells [16,17]. Retinoic acid is also essential for the expression of the key homing receptors, α4β7 and CCR9, on the surfaces of T and B cells [16,17]. The expression of α4β7 on B cells is dependent on retinoic acid [17] and is responsible for their migration in the intestine and secondary lymphoid organs by interacting with MAdCAM-1 [4]. In our prior study, the dam of VAD + VA + RVA piglets showed enhanced maternal systemic adaptive immunity characterized by increased RVA-specific IgA/IgG antibody-secreting cell numbers in blood, milk, and a number of other tissues, such as spleen, mammary gland, and ileum, as a possible impact of supplemental oral VA compared with the dam of VAD + RVA piglets [11]. Thus, we expected some possible difference in mean MAdCAM-1- or CCL25-positive scores in the turbinates of VAD + VA + RVA and VAD + RVA groups. In our study, however, MAdCAM-1- or CCL25-positive scores in the nasal turbinates of VAD + VA + RVA- and VAD + RVA-group pigs did not differ significantly (Table 1). These results might be associated with the lack of differences in serum IgA or IgG antibody titers between VAD + VA + RVA- and VAD + RVA-group pigs from PID 0 to 14 as described previously [11], as well as no differences in mean IgA- or IgG-positive cell scores in the turbinate mucosal epithelium or lamina propria between the two group pigs (Table 1).

A previous study reported the presence of pIgR in the crypts of the small intestines of pigs [9]. Under the prior authors’ IHC staining conditions, pIgR mostly appeared to be in the apical cytoplasm of the crypt epithelial cells. Our study clearly demonstrated that pIgR is also present in the nasal turbinates of pigs. Like localization of pIgR in the small intestine of pigs, pIgR was detected on the apical and/or basal surface of the epithelial cells lining the turbinate mucosal epithelium. Compared with pIgR in the epithelial cells forming the mucous glands, pIgR in the turbinate mucosal epithelial cells may be essential to transport pIgs such as dimeric IgA across the epithelial barrier into the lumen. Similar to the intestine, the secretion and release of pIgs into the lumen may occur following proteolytic cleavage in the turbinate mucosal epithelial cells [8].

## 5. Conclusions

Collectively, our study was first to reveal that low numbers of advillin-positive, bottle-shaped cells that morphologically resemble tuft cells were present in the mucosal epithelium and mucous duct or glands. By IF staining, there were moderate to large amounts of MAdCAM-1 and CCL25 in the endothelial cells lining the vascular structures present in the lamina propria of nasal turbinates and moderate to large amounts of pIgR in the mucosal epithelium. The IgA- or IgG-positive cells were also predominantly present in the lamina propria of porcine nasal turbinates. There were no differences in IF- or IHC-positive scores or positive cell numbers of all parameters tested between piglets of VAD + VA + RVA and VAD + RVA sows, suggesting few or no effects of VA in sows on the parameters tested in piglets. Our study demonstrated the use of IHC/IF staining methods for the detection of tuft cells, MAdCAM-1, CCL25, pIgR, and IgA- or IgG-positive cells in porcine nasal turbinates, expanding our understanding of the in situ distribution and comparison in the nasal cavity of pigs born to sows fed vitamin A-deficient diets with or without vitamin A supplementation.

## Figures and Tables

**Table 1:** Mean numbers (±SDM) of immunohistochemistry (IHC)-stained, advillin-positive tuft cells and mean (±SDM) IHC or immunofluorescence (IF) staining scores of mucosal vascular addressin cell adhesion molecule 1 (MAdCAM-1), chemokine ligand 25 (CCL25), polymeric immunoglobulin receptor (pIgR), and IgA- or IgG-positive cells in the nasal turbinates of conventional VAD + VA + RV piglets and VAD + VA piglets at PID 14 (15 to 17 days old).

IHC/IF		Regions Where IHC- or IF-Positive Cells Are Detected	Pigs ^a^	
			VAD + VA + RVA (*n* = 6) ^a^	VAD + RVA (*n* = 6) ^a^
IHC	Advillin ^b^	Mucosal epithelium	4.0 (2.6)	2.8 (2.8)
		Lamina propria (glandular epithelium)	0.4 (1.1)	0.3 (0.8)
IF	MAdCAM-1 ^c^	Mucosal epithelium	0 (0)	0 (0)
		Lamina propria	2.4 (0.4)	2.3 (0.8)
IF	CCL25 ^c^	Mucosal epithelium	0 (0)	0 (0)
		Lamina propria	2.6 (0.4)	2.4 (0.5)
IF	pIgR ^d^	Mucosal epithelium	2.1 (0.3)	2.1 (0.5)
		Lamina propria (glandular epithelium)	2.3 (0.4)	2.2 (0.5)
IHC	IgA-positive cells ^e^	Mucosal epithelium	0.1 (0.3)	0.2 (0.4)
		Lamina propria	2.6 (0.6)	2.5 (0.5)
IF	IgG-positive cells ^e^	Mucosal epithelium	0.3 (0.3)	0.2 (0.3)
		Lamina propria	2.6 (0.5)	2.5 (0.6)

^a^ Four rotavirus seropositive sows were fed vitamin A-deficient (VAD) diets at approximately gestation day (GD) 30. Two of four VAD sows (VAD + VA) were given daily oral retinyl palmitate (30,000 IU) starting at approximately GD 76 throughout the rest of gestation and lactation. All four sows were inoculated orally with 1 × 10^9^ FFU of rotavirus A (RVA) strain OSU G5[P7] at GD 84–93 [VAD + VA + RVA sows (*n* = 2); VAD + RVA sows (*n* = 2)]. Their 3–5-day-old piglets were also inoculated orally with 1 × 10^8^ FFU of the same rotavirus. Piglets were euthanized at approximately post-inoculation day (PID) 14. The intestinal tissues, liver, and blood were collected to investigate the impact of oral VA in maternal immunity of VAD sows and passive protection of their piglets against RVA [11]. In addition, the nasal turbinate (or nasoturbinate) tissues were also collected from three pigs per sow [VAD + VA + RV piglets (*n* = 6); VAD + VA piglets (*n* = 6)] at PID 14. ^b^ Mean numbers of advillin-positive tuft cells were estimated by directly counting IHC-positive cells in four different microscopic areas (×200) of a nasal turbinate section of each pig, and the mean (±standard deviation) of all group pigs’ values was calculated. ^c^ The MAdCAM-1 or CCL25 antigen-positive score per group was evaluated based on the mean number and intensity of IF-positive cells in three different microscopic areas (×200) of a nasal turbinate section of each pig, as follows: 0, no positive vessels showed staining; 1, low numbers of positive vessels showed mild staining; 2, moderate numbers of positive vessels showed moderate staining; and 3, high numbers of positive vessels showed strong staining. ^d^ The pIgR antigen-positive score per group was evaluated based on the mean number and intensity of IF-positive cells in three different microscopic areas (×200) of a nasal turbinate section of each pig, as follows: 0, no positive cells showed staining; 1, low numbers of positive cells showed mild staining; 2, moderate numbers of positive cells showed moderate staining; and 3, high numbers of positive cells showed strong staining. ^e^ The IgA- or IgG-positive score per group was evaluated based on the mean number of IHC- or IF-positive cells in three different microscopic areas (×200) of a nasal turbinate section of each pig, as follows: 0 (negative), no positive cells; 1 (low), less than 10 IHC- or IF-positive cells; 2 (moderate), 10 to 20 IHC- or IF-positive cells; and 3 (high), more than 20 IHC- or IF-positive cells.

## Data Availability

The original contributions presented in this study are included in the article. Further inquiries can be directed to the corresponding authors.

## Supplementary Materials

The following supporting information can be downloaded at https://www.mdpi.com/article/10.3390/vetsci13090987/s1, Figure S1: Hematoxylin and eosin-stained turbinate of pigs; Figure S2: RNAscope ISH-stained turbinate of pigs; Figure S3: Detection of advillin-positive tuft cells by IHC in porcine nasal turbinate tissues; and Figure S4: No detection of IgA- or IgG-positive cells by IHC or IF staining in the spleen of SCID gnotobiotic pigs.
