# Supplementary material for: Immunohistochemical Detection, In Situ Distribution, and Comparison of Tuft Cells and Other Selected Components of Mucosal Immunity in Nasal Turbinates of Piglets Challenged with Rotavirus from Sows Fed Vitamin A-Deficient Diets with or Without Vitamin A Supplementation
Source: Vet Sci. 2026 Sep 18;13(9):987. doi: 10.3390/vetsci13090987 (PMC13611996; doi:10.3390/vetsci13090987)
Supplement: Supplementary file 1 [file vetsci-13-00987-s001.zip › Supplementary Figures S1-S4.pdf]

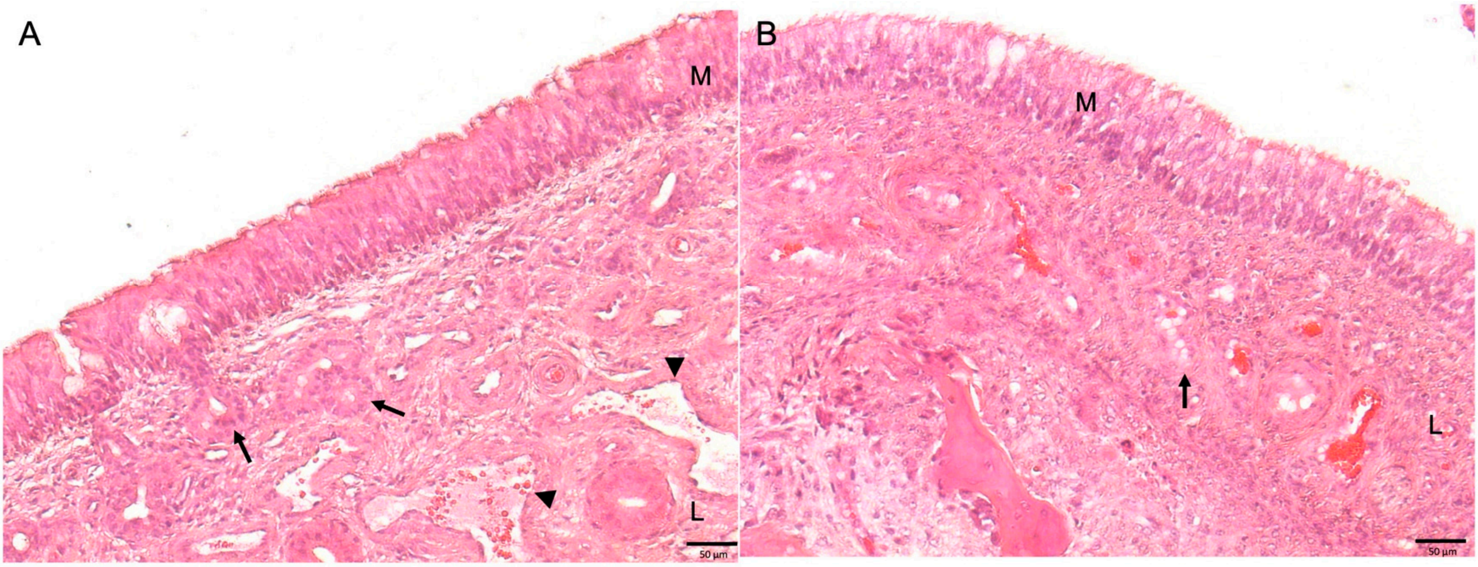

Supplementary Figure S1. Hematoxylin and eosin-stained turbinate of a pig from VAD+VA+RVA group (A) and a pig from VAD+RVA group (B) at post-inoculation day 14, showing no histological abnormalities. Note the normal mucosal epithelium mostly lined by pseudostratified ciliated columnar epithelial cells and fewer goblet cells; and the normal lamina propria composed of the connective tissues, mucous glands (arrows), and variable-caliber blood vessels (arrowheads). Scale bars = 50 µm. M, mucosal epithelium; L, lamina propria.

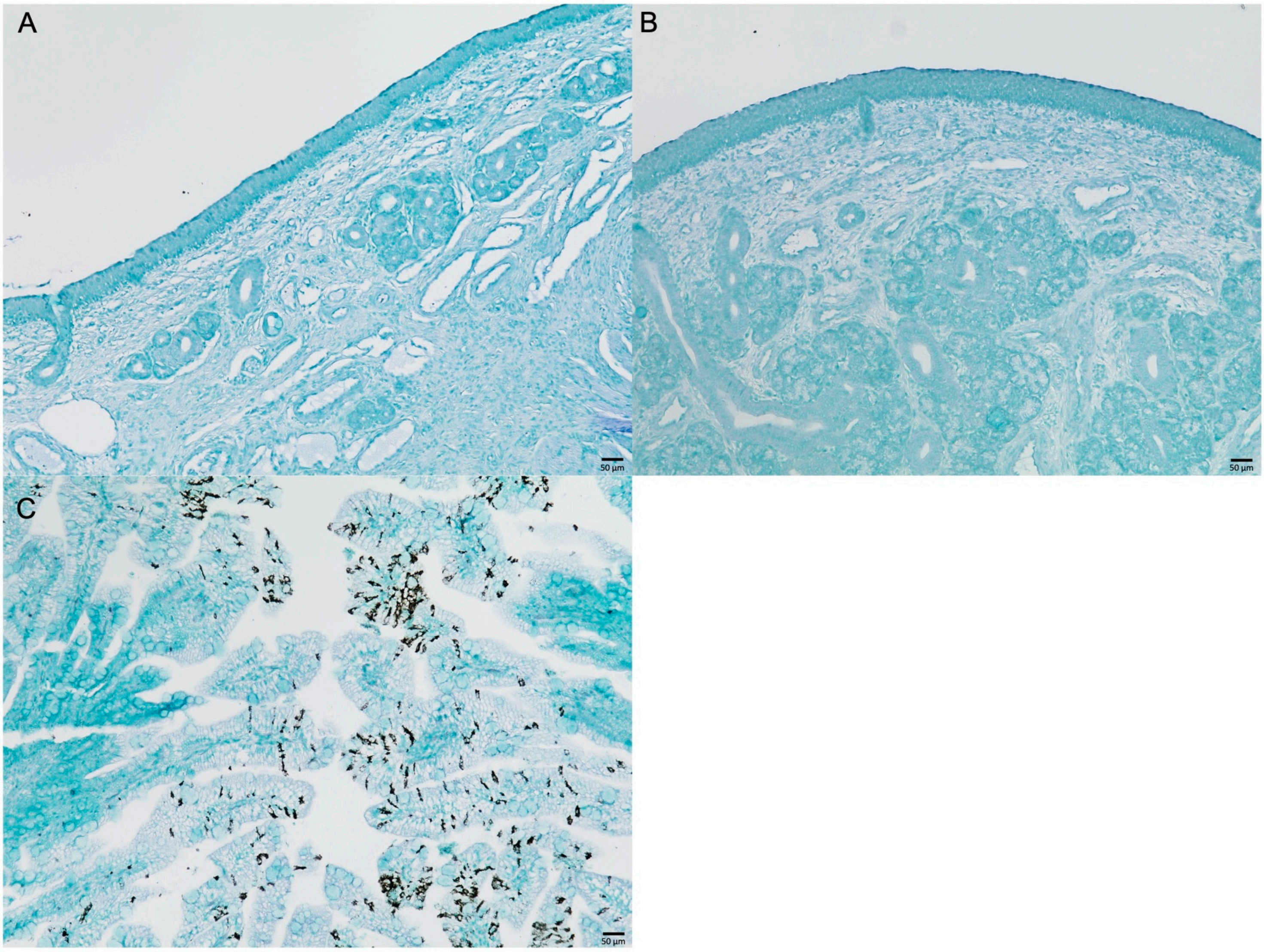

Supplementary Figure S2. RNAscope in situ hybridization (ISH)-stained turbinate of a pig from VAD+RVA group (A) and a pig from VAD+VA+RVA group (B) at post-inoculation day 14, showing no ISH-positive cells. (C) ISH-stained intestinal tissue of a rotavirus A-infected germfree pig used as a positive control, showing high numbers of ISH-positive cells (black color) in the villous epithelium. Scale bars = 50 μm. Methyl green counterstaining

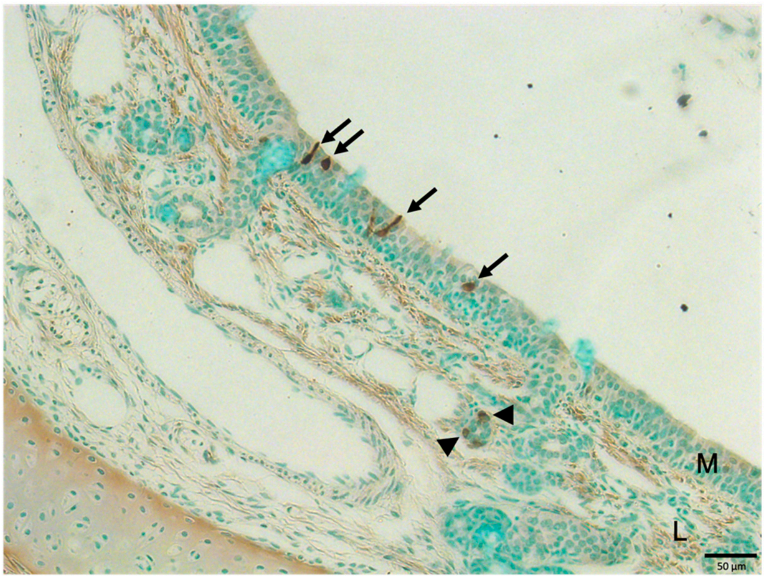

Supplementary Figure S3. Detection of advillin-positive tuft cells by immunohistochemistry (IHC) in porcine nasal turbinate tissues. IHC-stained turbinate of a pig from VAD+VA+RVA group at post-inoculation day 14, showing a low to moderate number of advillin-positive tuft cells (dark brown color) in the turbinate epithelium (arrows) or mucous gland (arrowheads). Note a few bottle- or round-shaped, IHC-positive cells in the turbinate epithelium or mucous gland. Scale bars = 50 µm. DAB substrate; methyl green counterstaining. M, mucosal epithelium; L, lamina propria.

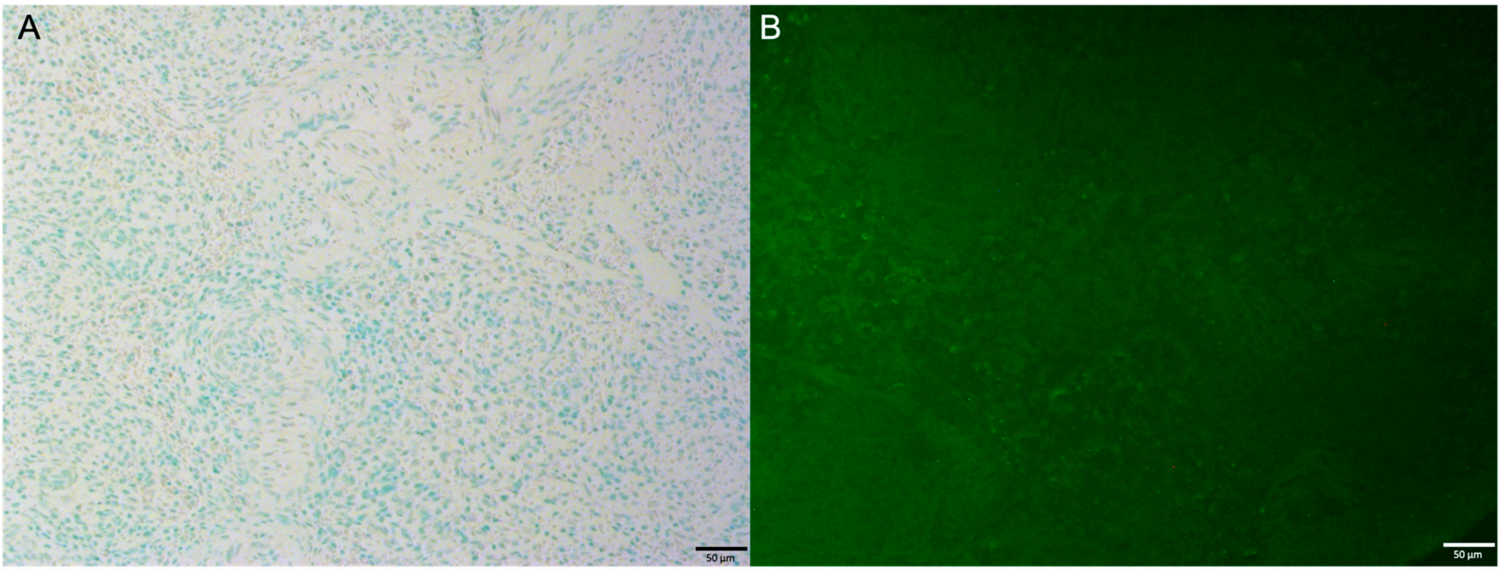

Supplementary Figure S4. No detection of IgA- (A) or IgG-positive cells (B) by immunohistochemistry (IHC) or immunofluorescence (IF) staining in the spleen of a Severe Combined Immunodeficiency (SCID) gnotobiotic pig deficient in B and T cells. (A) IHC-stained spleen of a SCID pig, showing no intrafollicular or extrafollicular IgA-positive cells. (B) IF-stained spleen of a SCID pig, showing no intrafollicular or extrafollicular IgG-positive cells. Scale bars = 50 µm. (A), DAB substrate and methyl green counterstaining.
